# Calcipotriol attenuates liver fibrosis through the inhibition of vitamin D receptor-mediated NF-κB signaling pathway

**DOI:** 10.1080/21655979.2021.2024385

**Published:** 2022-01-19

**Authors:** Jian Gong, HuanYu Gong, Yang Liu, XinLan Tao, Hao Zhang

**Affiliations:** a Department of Infectious Diseases, The Third Xiangya Hospital of Central South University, Changsha, P. R. China; b Department of Pathology, The Third Xiangya Hospital of Central South University, Changsha, P. R. China; c Department of Nephrology, The Third Xiangya Hospital of Central South University, Changsha, P. R. China

**Keywords:** Vitamin D receptor, liver fibrosis, hepatic stellate cells, calcipotriol, NF-κB

## Abstract

Liver fibrosis is an inevitable stage in the development of chronic liver disease to cirrhosis. Nonetheless, the interventional treatment and achieving control over the disease at this stage can substantially reduce the incidence of liver cirrhosis. To demonstrate these aspects, liver pathological sections of 18 patients with chronic liver disease are collected for research according to the degree of fibrosis. Further, the expressions of related proteins in each group are studied by the Western blot method. The cell proliferation and apoptosis are detected by CKK-8 and flow cytometry analyses. Further, a rat model with carbon tetrachloride (CCl_4_)-induced liver fibrosis is employed to verify the effect and mechanism of VDR on the process of liver fibrosis *in vivo*. The expression of VDR in liver tissues of patients with liver fibrosis is negatively correlated with α-smooth muscle actin (α-SMA), Col-1, and liver fibrosis stages. Moreover, the tumor necrosis factor (TNF)-α stimulation could increase the proliferation of LX-2, up-regulate the expression of α-SMA, Col-1, NF-κB, p-IκBα, p-IKKβ, p-p65m, and some fibrosis factors, as well as down-regulate the expressions of VDR and matrix metalloproteinase-1 (MMP-1). Considering the protective actions of VDR, calcipotriol, a VDR agonist, effectively reduced the degree of liver fibrosis in a rat model of liver fibrosis by inhibiting the deposition of extracellular (ECM) and activation of hepatic stellate cells (HSCs), which is negatively correlated with the degree of liver fibrosis. Together, these shreds of evidence demonstrated that the calcipotriol showed great potential in effectively attenuating liver fibrosis.

## GRAPHICAL ABSTRACT

**Figure:**
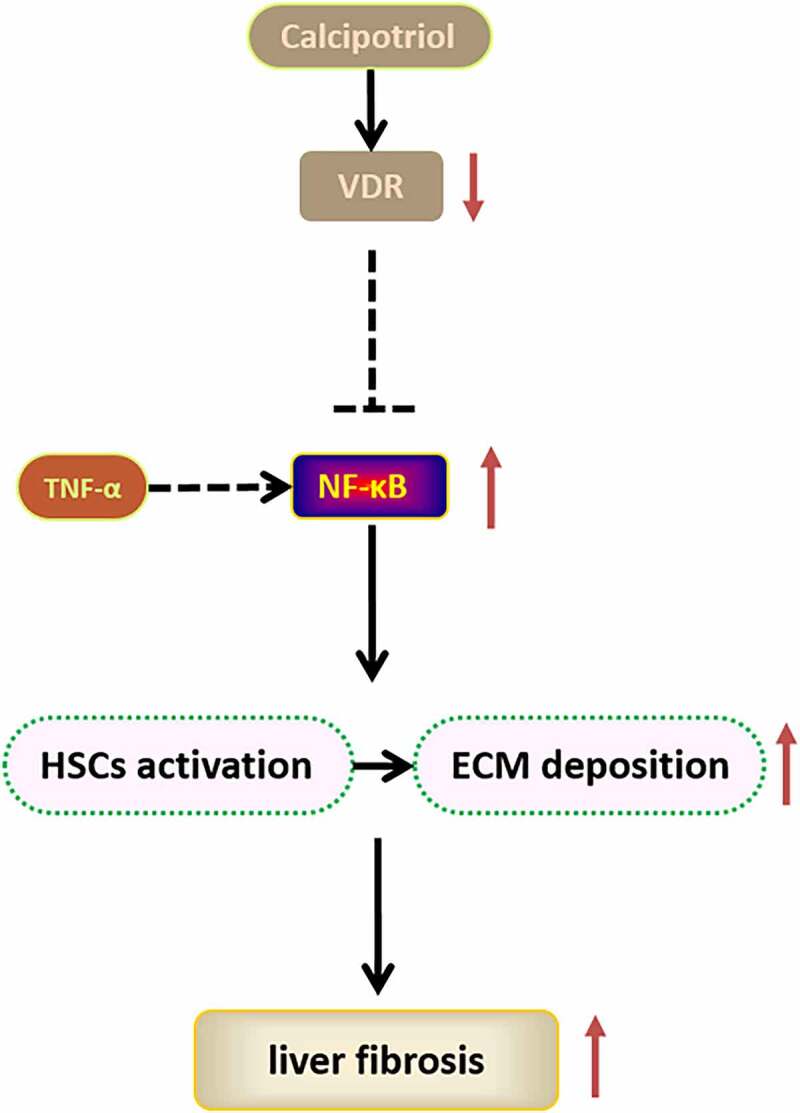

## 1. Introduction

Liver cirrhosis, late-stage liver damage, is a chronic, progressive, and diffuse liver disease caused due to various pathological conditions and physical activities, resulting in long-term damage of the liver. Notably, this pathological condition is often accompanied by portal hypertension and multiple complications in its late-stage [1,2]. Every year, liver cirrhosis affects hundreds of thousands of people globally [3]. Notably, liver fibrosis is an inevitable stage of the development of chronic liver disease into cirrhosis, which is the repair response after liver tissue injury. This repair response initially involves the activation of hepatic stellate cells (HSCs) and the maintenance of the dynamic balance of the extracellular matrix (ECM). In this context, the activation of HSCs further results in the secretion of a large amount of ECM, making the fibrous connective tissue proliferate diffusely. Finally, these consequences gradually worsen and result in the subsequent appearance of pseudo-lobules, leading to cirrhosis. Although several factors lead to liver cirrhosis, controlling the disease at the stage of liver fibrosis, such that the disease can be terminated or reversed, is a major topic in the current chronic liver disease research.

Considering liver fibrosis, the stimulation of inflammatory mediators is one of the most important causes of HSCs activation, which further produces a large amount of ECM and promotes the process of liver fibrosis. Under inflammation or other stimulating factors, a large amount of inflammatory factors is released, and HSCs in a resting state are activated. Then, the activated HSCs are transformed into myofibroblasts (MFB), which synthesize a large amount of collagen fibers. Among them, TNF-α, as an endogenous cytokine mainly produced by the monocyte-macrophage system, can participate in inflammation through NF-κB and other pathways, thereby activating HSCs [4,5]. In addition, the activation of HSCs involves a variety of cytokines and signal transduction pathways, such as platelet-derived growth factor (PDGF), transforming growth factor β1 (TGF-β1), matrix metalloproteinase-1 (MMP-1), and tissue inhibitor of matrix metalloproteinases-1 (TIMP-1), among others. The imbalance of MMP-1/TIMP-1 expressions in the activated HSCs may be an essential factor, leading to subsequent ECM deposition.

Vitamin D receptor (VDR), a member of the steroid hormone/thyroid hormone superfamily, can be classified into two categories: 1) membrane receptor (mVDR) and 2) nuclear receptor (nVDR), expressed in almost all nucleated cells [6]. mVDR is primarily responsible for the balance of calcium and phosphorus toward maintaining normal bone tissue metabolism. In contrast, nVDR is essentially a nuclear transcription factor-dependent on its ligand (active vitamin D3). More often, Vitamin D regulates the expression of structural genes mainly due to its effect on the nVDR in its target cells to form a hormone-receptor complex. Indeed, it is transported into the nucleus and binds to the promoter region of the relevant target genes, thereby affecting the transcription of the genes. Recent studies indicated that VDR as a nuclear transcription factor was widely involved in the proliferation and differentiation of cells, generating immune-inflammatory responses, and fibrosis regulation [7,8]. The anti-inflammatory effect of VDR has a significant protective effect on the kidney and cardiovascular. Moreover, its mechanism could be related to the inhibition of the VDR-mediated NF-κB signaling pathway [9–11]. In this vein, our group has demonstrated that the down-regulation of VDR was closely related to the severity of the proteinuria of a patient with type-2 diabetes [12]. It was observed that the expression of VDR in the kidney of unilateral ureteral obstruction (UUO) model was significantly reduced, indicating its relevance to renal fibrosis [13]. In animal models of pulmonary fibrosis, active vitamin D3 (VitD3) has presented an inhibitory effect on the occurrence and development of pulmonary fibrosis [14]. In the rat IPF model, active VitD3 attenuated the concentration of calcium ions in alveolar type II epithelial cells and inhibited the PI3K-AKT-mTOR pathway, thereby inhibiting the occurrence and development of IPF [15]. In addition, several reports demonstrated that vitamin D supplementation could be of great significance in delaying the progression of liver cirrhosis [16]. As a receptor for active vitamin D, the expression of VDR in liver tissue and its relationship with liver fibrosis are worthy of our further study.

In the human body, the liver is the critical metabolic site of vitamin D. Moreover, the active vitamin D can inhibit liver fibrosis to a certain extent by inhibiting the activation of HSCs. In this context, several reports demonstrated that 1,25(OH)2D3 could significantly inhibit the proliferation of LX-2 cells and promote their apoptosis. Moreover, it was demonstrated that 1,25(OH)2D3 could inhibit the activation of LX-2 cells by TGF-β1 [17]. In the CCl_4_-induced liver fibrosis model, 1,25(OH)2D3 showed a pronounced anti-hepatic fibrosis effect through inhibiting the activation of HSCs and the expression of related fibrosis markers [18]. In a TAA-induced mouse liver fibrosis model, 1,25(OH)2D3 also presented prominent anti-liver fibrosis [19]. Notably, the anti-hepatic fibrosis effect of vitamin D is closely related to its receptor, i.e., VDR. Calcipotriol, a selective VDR agonist, offers a specific anti-hepatic fibrosis effect in animal models with liver cirrhosis [20]. In addition, VitD3 is closely related to chronic liver disease. The lowered serum active vitamin D level caused by the damage of liver tissue structure and function in patients with liver cirrhosis is related to the severity of liver disease [21]. Moreover, there exist reports that VitD3 could inhibit proliferation and induce apoptosis in tumor cell lines and human HSCs and reduce the degree of CCl_4_-induced liver fibrosis in rats [22–24]. However, the large intake of vitamin D can cause abnormal calcium and phosphorus metabolism, increase the blood calcium content, and trigger other biological reactions, limiting the clinical application of vitamin D toward anti-tumor and anti-fibrosis effects [25]. Considering these aspects, it is worth investigating that VDR, which is the receptor of VitD3, is related to liver fibrosis and presents its clinical application potential.

In this study, we hypothesized that the differential expression of VDR is implicated in the progression of liver fibrosis by regulating the activation of HSCs. Based on the liver tissue pathological specimens of patients with liver fibrosis, we detected the correlation between the expression of VDR, α-SMA, as well as Col-1 and the degree of liver fibrosis. In addition, the effect of the expression of VDR and its agonist calcipotriol on HSCs activation and the mechanistic views were studied using the LX-2 cell model. Finally, we used the CCl_4_-induced rat liver fibrosis model to explore the anti-liver fibrosis effects of VDR and calcipotriol. Accordingly, we aimed to systematically study the effect of VDR on liver fibrosis and the possible mechanisms for regulating liver fibrosis and provide a potential target for the treatment of liver fibrosis.

## 2. Materials and methods

### 2.1. Sample collection

Initially, the pathological liver specimens were collected from patients (18 cases) with chronic liver disease who were admitted to the Department of Pathology, Xiangya Third Hospital of Central South University from 2017.05 to 2018.05. Further, the specimens were divided into 2 groups according to the grade of fibrosis using the Metavir scoring system by two pathologists: mild liver fibrosis group (F1-F2) for 10 cases and severe liver fibrosis group (F3-F4) for 8 cases. The patients’ informed consent has been obtained for the above cases, and the related trials have been approved by the ethics committee.

### 2.2. Cell culture and transfection

The human hepatic stellate cell line, LX-2, used in this study was purchased from the National Collection of Authenticated Cell Cultures. The cells were cultured in Dulbecco’s modified Eagle’s medium (DMEM, Gibco, Grand Island, USA) containing 10% fetal bovine serum (FBS, Gibco) and placed in an incubator at 37°C and 5% CO_2_. In the calcipotriol treatment experiment, when the fusion degree of the cultured cells was about 70–80%, calcipotriol (10^−7^moL/L, Shanghai Yubo Company, Shanghai, China) was used to stimulate the cells for 24 h. To study the mechanism, based on the pGPU6/GFP/Neo vector, the stably transfected small heparin ribose nucleic acid (shRNA) plasmids (VDR-ShRNA 1337, VDR-ShRNA 824, VDR-ShRNA 649, and VDR-ShRNA 1805) were constructed, and their target sequences were as follows: 5ʹ-GCTGAAGTCAAGTGCCATTGA-3ʹ, 5ʹ-GGAGTTCATTCTGACAGATGA-3ʹ, 5ʹ-GCTTTCACTTCAATGCTATGA-3ʹ, 5ʹ-GCTCGAAGTGTTTGGCAATGA-3ʹ. The transfection experiment was carried out according to the manufacturer’s instructions of Lipofectamine™ 2000 (Wuhan Kehaojia Biological Technology Co., Ltd., Wuhan China).

### 2.3. RNA extraction and reverse transcription-quantitative polymerase chain reaction (RT-qPCR)

TRIzol (Invitrogen, Camarillo, USA) was added directly to the 35 mm dish to lysis the cells. The subsequent operations were carried out according to the instructions of the TRIzol as previously described [22]. Further, the concentration of total RNA was detected. Total RNA was reverse transcribed into complementary deoxyribose nucleic acid (cDNA) by the reverse transcription kit (Fermentas). The qPCR was carried out using SYBR GreenPCR MasterMix (ABI) based on the Applied Biosystems 7500 detection system (ABI). The 2^−ΔΔCt^ method was used to calculate the relative expression of VDR in each group with the GAPDH gene as an internal reference. The primers used in the experiment were synthesized by Takara. The sequence is as follows: VDR: Forward Primer: 5ʹ-GGCCGGACCAGAAGCCTTT-3ʹ, Universal Adaptor PCR Primer: 5ʹ-GGTGAATAGTGCCTTCCGCT-3ʹ; GAPDH: Forward Primer: 5ʹ- CAATGACCCCTTCATTGACC-3ʹ; Universal Adaptor PCR Primer: 5ʹ-GACAAGCTTCCCGTTCTCAG-3ʹ.

### 2.4. Cytoplasmic and nuclear fractionation assay

NucBuster TM Protein Exaction Kit (Merck, NJ, USA) was used to extract nuclear proteins from cells. Briefly, the cells were initially collected in a centrifuge tube and ground into a powder with liquid nitrogen. Then, about 150 µl of NucBuster Reagent 1 was added, mixed thoroughly, and centrifuged at 16,000 g at 4°C for 5 min. The supernatant of cytosolic protein components was used to detect internal controls or other cytoplasmic proteins. Subsequently, a mixture containing 1 μL of 100× Protease Inhibitor Cocktail solution, 1 μL of 100 mM DTT, and 75 μL of NucBuster Exaction Reagent 2 solution, was sequentially added to the precipitate, mixed thoroughly, and then centrifuged at 16,000 g at 4°C for 5 min. The supernatant was indicated as the nucleoprotein solution.

### 2.5. Western blot assay

Initially, the lysis solution (Beyotime Biotechnology, Haimen, Jiangsu) was added to the cell culture dish to extract the complete protein, as described previously [22]. Briefly, tissue protein was extracted by shredding and treating with the lysate to make a homogenate. The homogenate was transferred to a clean centrifuge tube and centrifuged at 12,000 rpm for 10 min. Then, the supernatant was transferred to a new centrifuge tube containing the total protein. Further, the total protein of each group was separated by electrophoresis by SDS-PAGE and transferred to polyvinylidene fluoride (PVDF) membrane. It was blocked using 5% skimmed milk at room temperature for 1 h. Then, the primary antibody rabbit Col-1 antibody (Abcam, Cambridge, UK), rabbit VDR antibody (Abcam), rabbit TGF-β1 antibody (Abcam), rabbit desmin antibody (Abcam), rabbit IL-6 antibody (Abcam), rabbit CTGF antibody (Abcam), rabbit α-SMA antibody (Abcam), mouse PDGF antibody (Abcam), mouse MMP-1 antibody (Abcam), rabbit TIMP-1 antibody (Abcam), rabbit Bcl-2 antibody (CST), rabbit Bax antibody (CST), rabbit cytochrome-c antibody (CST), rabbit Histone H2 antibody (Abcam), rabbit NF-κB antibody (Abcam), rabbit p-IκBα antibody (Abcam), rabbit p-IKKβ antibody (Abcam), rabbit p-p65antibody (Abcam), rabbit IκBα antibody (Abcam), rabbit IKKβ antibody (Abcam), rabbit p65antibody (Abcam), and mouse GAPDH antibody (Santa Cruz, CA, USA) were incubated with membrane overnight at 4°C. Subsequently, the secondary antibody (Abcam) labeled with horseradish peroxidase (HRP) was incubated at 37°C for 1 h. Finally, the Pierce™ electrochemiluminescence (ECL) Plus Western blotting Substrate (Thermo Scientific, Waltham, USA) was used to expose the protein band on the X-ray Film for Western blot Detection (Thermo Scientific) instrument. The gray value of the band was measured by Quantity One software. GAPDH was used as an internal control.

### 2.6. Immunohistochemistry (IHC)

The clinically collected pathological sections of liver tissues were carried out using a process of baking slices, dewaxing, and antigen retrieval and used for immunohistochemical staining. First, the tissue sections were blocked with goat serum blocking solution, placed in a humid chamber, and incubated at 37°C for 30 min. Then, the well-mixed VDR, α-SMA, and Col-1 primary antibody diluent were added and incubated at 4°C overnight. Subsequently, it was placed in a 37°C incubator and incubated for 45 min. After washing, HRP-labeled secondary antibody was added and incubated at 37°C for 1 h. After washes, DAB dye (DAB1:DAB2 diluted in a ratio of 1:20, Beijing Zhongshan Golden Bridge Biotechnology Co., Ltd. Beijing, China) was added to dye for 5 min in the dark. Finally, after counterstaining with hematoxylin and dehydrating with ethanol, the slides were observed and evaluated under a microscope (OLYMPUS, Tokyo, Japan).

### 2.7. Cell proliferation assay

The density of the cell suspension was adjusted to 20,000 cells/ml with culture medium and seeded into a 96-well plate at about 100 µl/well. Each group had 5 replicate wells. After the cells adhered to the plate, 10 μL of cell counting kit (CCK)-8 solution (Beyotime Biotechnology) was added to each well and incubated for 4 h. Finally, the OD value at 450 nm wavelength was measured and recorded using the microplate reader (Shanghai Kehua Bio-Engineering Co., Ltd. Shanghai, China), and the cell growth curve was plotted with time as the abscissa and OD value as the ordinate.

### 2.8. Enzyme-linked immunosorbent assay (ELISA)

The Col-1 ELISA quantitative kit (Shanghai Haring Biological Technology Co., Ltd. Shanghai, China) was used to detect the content of Col-1 in the cell supernatant. Briefly, the standard solution was initially prepared and tested on the microtiter plate by setting the blank, standard, and sample wells, respectively. Then, the sample was added accordingly and incubated at 37°C. Finally, 50 μL of enzyme-labeled reagent was added and incubated for 30 min. The OD value at 450 nm wavelength was measured and recorded on the microplate reader, and the blank sample was adjusted to zero. The standard curve was plotted with the standard concentration as the abscissa and the OD value as the ordinate.

### 2.9. Cell apoptosis assay

Initially, about 5 × 10^5^ cells were collected and washed with PBS. Then, 500 μL of binding buffer was added and tapped gently to suspend the cells. Then, 5 μL of Annexin V-FITC and 5 μL of propidium iodide (PI) were added and mixed with the cell suspension and incubated at room temperature in the dark for 15 min. Finally, the cell suspension was detected using the BD FACSAria II flow cytometer (Becton, Dickinson, and Company) to calculate the apoptosis rate.

### 2.10. Animal model

A total of 15 Sprague Dawley (SD) rats (male, 7-week-old) were selected, and their body weights were maintained between 190 and 220 g. Rats were allocated to each group randomly, food and water were freely available. Rat liver fibrosis model was induced by CCl_4_ subcutaneous injections (3 mL/kg, CCl_4_ diluted with vegetable oil) biweekly for 8 weeks. Based on the same dose of CCl_4_ in the model group, the calcipotriol dissolved in vegetable oil was started by gavage every day at a dose of 20ug/kg from the fourth week until the 8^th^ week. The rats in each group were weighed once a week. At the end of the 8^th^ week, the rats were sacrificed, and the abdominal aortic blood (about 5 mL) was drawn out and collected into a sterile centrifuge tube. The liver was separated entirely and weighed, as well as a thin section of each separated liver was fixed with 10% paraformaldehyde (for 7 days) and routinely dehydrated, embedded, and sectioned. All the related feeding and handling of rats comply with the relevant regulations of China’s ‘Experimental Animal Management Regulations’.

### 2.11. HE staining

HE staining of the dissected tissue sections was performed as described previously [22]. Briefly, After the tissue sections were routinely deparaffinized and rehydrated, they were stained with hematoxylin for 3 min. Further, after soaking in 0.5% hydrochloric acid-alcohol for 30 s, it was treated with gradient alcohol and stained with 0.5% eosin for 1 min. Then, after dehydration with gradient alcohol and transparent with xylene, the slides were sealed with neutral gum. Finally, the slides were observed under the microscope (OLYMPUS).

### 2.12 Masson staining

After the tissue sections are routinely deparaffinized and rehydrated, they are stained with Weigert iron hematoxylin staining solution for 5–10 min. Then, acidic ethanol differentiation solution was dripped onto the sliced tissue for 5–15s. After a wash, Masson blue solution was added dropwise on the tissue section to return to blue for 3–5 min. Then, Masson Ponceau Magenta staining solution was added dropwise for 5–10 min. Then, after washing the sections with a weak acid working solution, 1% phosphomolybdic acid aqueous solution was added dropwise to differentiate the tissues for 3–5 min and placed in the aniline blue solution for staining for 2 min. Finally, after dehydration with gradient alcohol and transparent with xylene, the slides were sealed with neutral gum and were observed under the microscope (OLYMPUS).

### 2.13. Data analysis

The SPSS 16.0 statistical software was used to analyze the data. The rank-sum test in the non-parametric test was used for statistical analysis, and the correlation analysis was performed by Spearman rank correlation analysis. The data comparison between the two groups was performed by t-test, and the comparison among multiple groups was calculated by One-way analysis of variance (ANOVA), followed by the post-hoc LSD method. The Welch method was used for the test if the variance was uneven, and the Dunnett’s T-test was used for the pairwise comparison. All experimental data are the mean ± SD of at least three independent experiments. *P < 0.05, **P < 0.01, and ***P < 0.001.

## 3. Results

In this study, we hypothesized that the differential expression of VDR is implicated in the progression of liver fibrosis by regulating the activation of HSCs. Based on the CCl_4_-induced rat model of liver fibrosis and TNF-α stimulated cell model of HSCs, we demonstrated that VDR regulated progression of liver fibrosis and cell function of HSCs with the participation of NF-κB signaling pathway. In this approach, calcipotriol, a VDR agonist, was employed to effectively reduce liver fibrosis in rat and cell models of liver fibrosis.

### 3.1 Differential expression and correlation analysis of VDR in patients with liver fibrosis

**Figure 1.**
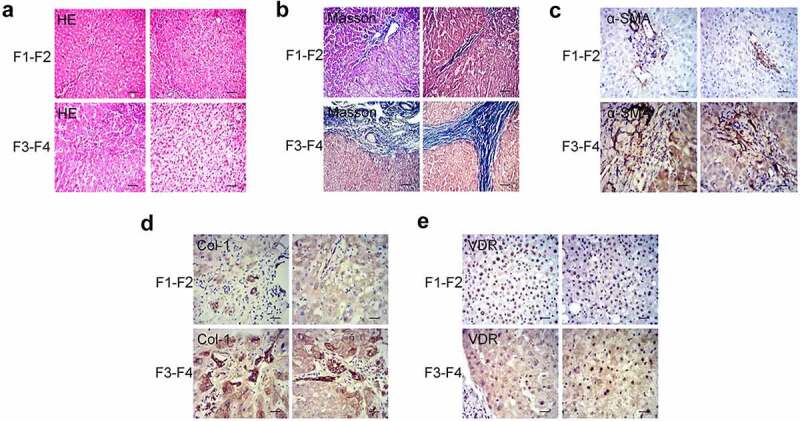
Differential expression and correlation analysis of VDR in patients with liver fibrosis. (a) and (b) Pathology observation of HE and Masson staining of human liver tissue from patients with liver fibrosis (F1-F2: mild liver fibrosis; F3-F4: severe liver fibrosis). (c) and (d) Pathology observation of IHC staining for α-SMA and Col-1 of human liver tissue. (e) The expression of VDR in human liver tissue was detected by IHC staining. Scale bar, 100 μm (×100 magnification).

Initially, we have collected pathological liver specimens from patients (n = 18) with chronic liver disease and divided them into 2 groups according to the grade of fibrosis: mild liver fibrosis group (n = 10, F1-F2) and severe liver fibrosis group (n = 8, F3-F4) (Figure 1(a,b)). The results of immunohistochemistry showed that, regardless of the degree of liver fibrosis, α-SMA was expressed in the central vein wall and the portal area vein wall. Moreover, the expression of α-SMA in the severe liver fibrosis group was significantly stronger than that in the mild liver fibrosis group (Figure 1(c)). Similarly, the expression of Col-1 in the severe liver fibrosis group was significantly higher than that in the mild liver fibrosis group (Figure 1(d)). In addition, we tested the expression of VDR in liver tissues. It was observed that the expression of VDR in the cytoplasm and nucleus was more pronounced. Moreover, the expression of VDR in the mild liver fibrosis group was significantly stronger than that in the severe liver fibrosis group (Figure 1(e)). Spearman correlation analysis showed that the expression of VDR was negatively correlated with the expression of SMA and Col-1 and the stage of fibrosis in liver tissue (Table 1).

**Table 1.** Spearman correlation analysis of VDR and SMA, Col-1 and the stage of fibrosis

			VDR	SMA	COL	F
Spearman’s rho	VDR	Correlation Coefficient	1.000	−0.559*	−0.828**	−0.770**
		Sig. (2-tailed)	/	0.016	0.000	0.000
		N	18	18	18	18
	SMA	Correlation Coefficient	−0.559*	1.000	0.413	0.728**
		Sig. (2-tailed)	0.016	/	0.089	0.001
		N	18	18	18	18
	COL	Correlation Coefficient	−0.828**	0.413	1.000	0.720**
		Sig. (2-tailed)	0.000	0.089	/	0.001
		N	18	18	18	18
	F	Correlation Coefficient	−0.770**	0.728**	0.720**	1.000
		Sig. (2-tailed)	0.000	0.001	0.001	/
		N	18	18	18	18

*Correlation is significant at the 0.05 level (2-tailed)

**Correlation is significant at the 0.01 level (2-tailed)

The expression correlation of VDR and SMA, Col-1, or the stage of fibrosis was analyzed in liver tissue.

### 3.2 Effect of differential expression of VDR on the proliferation of LX-2 cells

**Figure 2.**
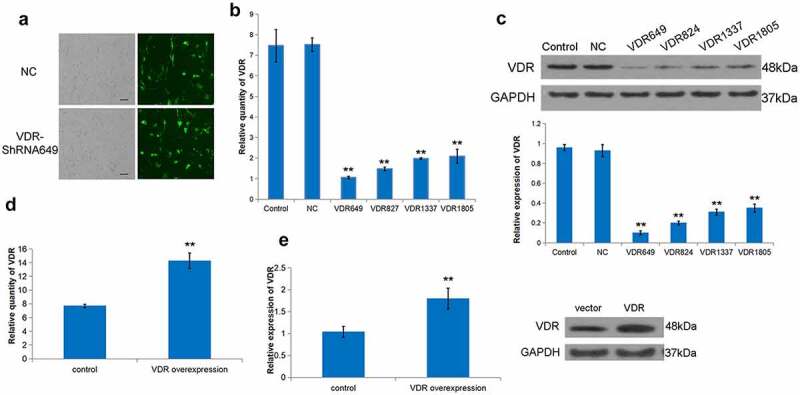
Effect of differential expression of VDR on the proliferation of LX-2 cells. (a) To evaluate the transfection rate of NC or VDR-shRNA, transfected LX-2 cells were shown through detecting GFP fluorescence. Scale bar, 50 μm. (b) and (c) The expression of VDR was detected by RT-qPCR and Western blot for multiplex VDR-shRNA screening. (d) and (e) Overexpression efficacy of VDR was validated in LX-2 cells by RT-qPCR and Western blot. (f) The effects of VDR overexpression or knockdown on the proliferation of LX-2 cells were detected by the CCK8 assay.

To further explore the role of VDR in liver fibrosis, a stable shVDR cell line was constructed based on the human hepatic stellate cell line LX-2 (Figure 2(a)). Further, VDR-ShRNA649 was screened as the highest knockdown efficiency group (Figure 2(b,c)). In addition, we constructed an effective VDR overexpressed LX-2 and applied it to the follow-up research (Figure 2(d,e)). Subsequently, we tested the effect of cells with differentially expressing VDR on their proliferation ability. It was evident that the cell proliferation ability of the low VDR expression group was stronger than that in the VDR high expression group (Figure 2(f)).

### 3.3 VDR regulates the expression of fibrosis-related factors through the NF-κB pathway to affect liver fibrosis

**Figure 3.**
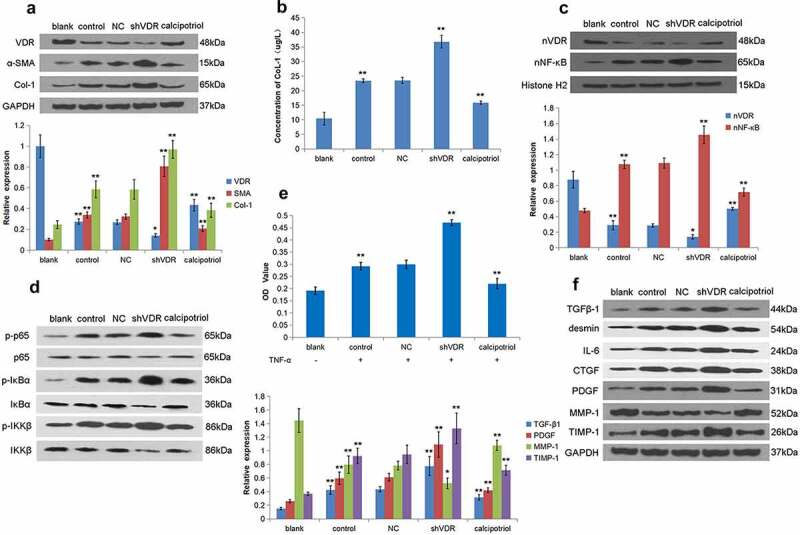
VDR regulates the expression of fibrosis-related factors through the NF-κB pathway to affect liver fibrosis. (a) The protein expression of VDR, α-SMA, and Col-1 in TNF-α-stimulated LX-2 cells following VDR knockdown or calcipotriol treatment were detected by Western blot. (blank: blank control group with conventional culture; control: experimental control group with TNF-α stimulation; NC: negative control group with TNF-α stimulation and transfection with shRNA-NC plasmid; shVDR: TNF-α stimulation and transfection with an shRNA-649 plasmid for knockdown VDR; calcipotriol: co-stimulated with TNF-α and calcipotriol). (b) The Col-1 content in the cell supernatant was validated by ELISA. (c) The expression of nVDR and nuclear NF-κB in TNF-α-stimulated LX-2 cells following VDR knockdown or calcipotriol treatment was detected by the Western blot. (d) The expression of p-IκBα, IκBα, p-IKKβ, IKKβ, p-p65 and p65 in TNF-α-stimulated LX-2 cells following VDR knockdown or calcipotriol treatment were detected by the Western blot. (e) The effects of VDR knockdown or calcipotriol treatment on the proliferation of TNF-α-stimulated LX-2 cells detected by CCK8 assay. (f) The expression of TGFβ-1, desmin, IL-6, CTGF, PDGF, MMP-1, and TIMP-1 in TNF-α-stimulated LX-2 cells following VDR knockdown or calcipotriol treatment were detected by Western blot.

TNF-α stimulation can activate LX-2 cells to construct a cell model of liver fibrosis. Accordingly, we detected the expression of VDR, α-SMA, and Col-1 in LX-2 cells stimulated by TNF-α. The results showed that the expression of VDR in the TNF-α stimulation group was lower than that of the control group, and the expression of VDR was significantly increased in the calcipotriol treatment group (Figure 3(a)). Notably, the expressions of α-SMA and Col-1 were increased in the shVDR group and decreased in the calcipotriol group (Figure 3(a)). At the same time, we also applied the ELISA method to detect the Col-1 content in the cell supernatant, and the results showed that its trend was consistent with the expression of Col-1 protein (Figure 3(b)). Our further study confirmed that the expression of nVDR was significantly increased in the calcipotriol group compared with the control group (Figure 3(c)). In addition, it was observed that the expression of nuclear NF-κB in the shVDR group was increased compared with the NC group, while the expression of nuclear NF-κB in the calcipotriol group was significantly lower than that in the control group and the shVDR group (Figure 3(c)). To further determine the VDR regulation of the expression of fibrosis-related factors through the NF-κB pathway, we detected the changes of p-IκBα, p-IKKβ, and p-p65 relative to their corresponding total proteins. However, the changes in their expression levels were consistent with the trend of the NF-κB pathway (Figure 3(d)). In addition, the effects of differential expression of VDR on cell proliferation were determined based on the TNF-α-stimulated LX-2 cell model. It was evident from the results that TNF-α stimulation resulted in enhanced cell proliferation compared with the control group, while upon TNF-α stimulation, the cell proliferation ability of the shVDR group was also higher than that of the NC group, while the cell proliferation ability of the calcipotriol group was reduced (Figure 3(e)). Furthermore, we tested the effect of TNF-α activated LX-2 cells on the relative expressions of various fibrosis-related factors, such as TGFβ-1, desmin, IL-6, CTGF, PDGF, MMP-1, and TIMP-1, in the case of differential expression of VDR. The results showed that TNF-α stimulation and VDR knockdown resulted in increased expression of TGFβ-1, desmin, IL-6, CTGF, PDGF, and TIMP-1, compared with the control group, while the expression of MMP-1 was reduced. Moreover, the expression of TGFβ-1, desmin, IL-6, CTGF, PDGF, and TIMP-1 was increased in the calcipotriol group, while the expression of MMP-1 was increased (Figure 3(f)).

### 3.4 VDR suppresses liver fibrosis by regulating cell apoptosis

**Figure 4.**
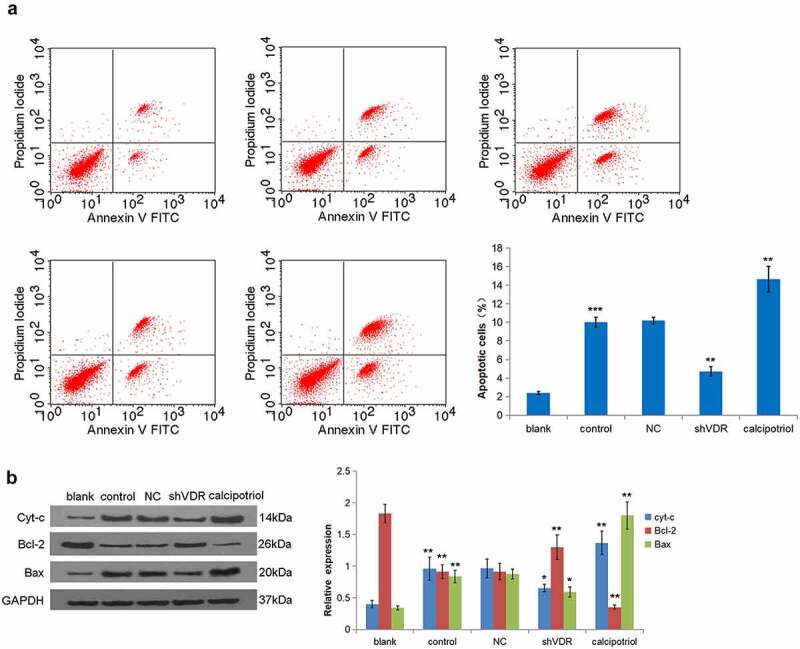
VDR suppresses liver fibrosis by regulating cell apoptosis. (a) The effects of VDR knockdown or calcipotriol treatment on the apoptosis of TNF-α-stimulated LX-2 cells were detected by flow cytometry. (b) The expression of Cyt-c and Bax in TNF-α-stimulated LX-2 cells following VDR knockdown or calcipotriol treatment were detected by Western blot.

HSCs apoptosis is closely related to liver fibrosis. The flow cytometry analysis demonstrated that the TNF-α stimulation promoted the apoptosis of LX-2 cells, while knockdown of VDR led to a decrease in the rate of apoptosis. Moreover, the calcipotriol treatment presented an increase in the rate of apoptosis (Figure 4(a)). In addition, the relative expression of key molecules in the mitochondrial apoptotic pathway was observed. It was evident that TNF-α stimulation increased the expressions of Cyt-c and Bax proteins and decreased the expression of Bcl-2. In contrast, the knockdown of VDR resulted in the expression of Cyt-c, whereas the expressions of Bax and Bcl-2 were different. Notably, the expressions of the notified proteins were completely opposite after the calcipotriol treatment (Figure 4(b)). Together, these relative expressions of various proteins were consistent with the changes in cell apoptosis.

### 3.5. VDR suppresses liver fibrosis in vivo

**Figure 5.**
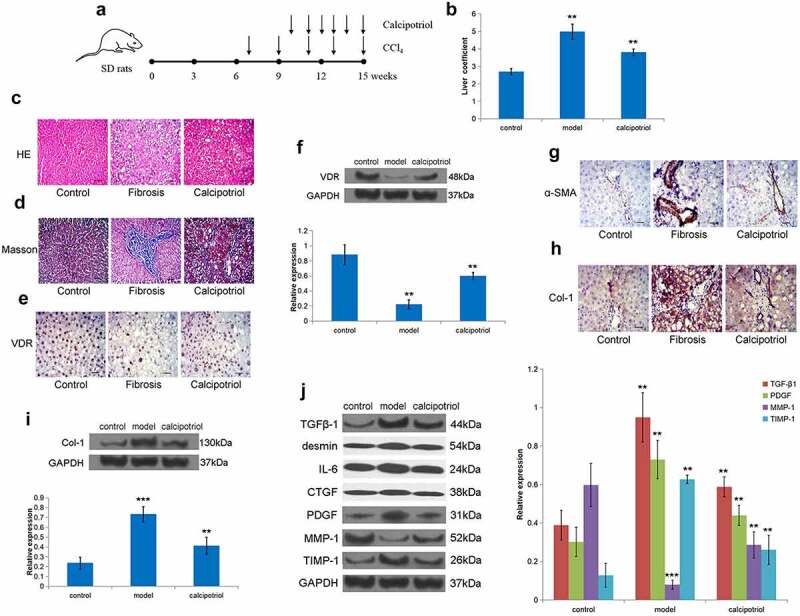
VDR suppresses liver fibrosis in vivo. (a) Schematic representation of CCl4-induced fibrosis model and reversion with calcipotriol. 7-week-old mice were intraperitoneally injected with CCl_4_ (3 mL/kg) biweekly for 8 weeks. Calcipotriol was administered via oral gavage every day at a dose of 20ug/kg from the 4^th^ week until the 8^th^ week. (b) The liver coefficient of the CCl_4_-induced liver fibrosis model with indicated treatment was evaluated. (c) and (d) Pathology observation of HE and Masson staining of liver tissue from rats liver fibrosis model to evaluate the degree of liver fibrosis in each group. (e-i) The expression of VDR, α-SMA, and Col-1 in liver tissue from rats liver fibrosis model with or without calcipotriol treatment were detected by Western blot and IHC. (j) The expression of TGFβ-1, desmin, IL-6, CTGF, PDGF, MMP-1, and TIMP-1 in liver tissue from rats liver fibrosis model with or without calcipotriol treatment were detected by Western blot. Scale bar, 100 μm (×100 magnification).

To further verify the effect of VDR on liver fibrosis *in vivo*, we constructed an SD rat liver fibrosis model through CCl_4_ treatment (Figure 5(a)). Through liver coefficient evaluation, it was observed that the liver coefficient of the model group was significantly higher than that of the control group, while the liver coefficient of the calcipotriol treatment group was significantly lower than that of the model group showing a certain therapeutic effect (Figure 5(b)). Moreover, the HE and Masson stainings showed that there was apparent fibrous tissue formation in the liver tissue of the model group, accompanied by regenerating nodules, indicating that the liver fibrosis model was successful, and calcipotriol treatment improved liver fibrosis to varying degrees (Figure 5(c,d)). Furthermore, we detected in rat liver tissue that the expression of VDR in the model group was significantly lower than that in the control and calcipotriol groups (Figure 5(e,f)). At the same time, the expression of α-SMA and Col-1 in the model group was also significantly increased, while the calcipotriol treatment significantly reduced the expression of α-SMA and Col-1 in the fibrosis model, indicating the reversal and alleviation of fibrosis (Figure 5(g-i)). Finally, it was observed that the expressions of TGFβ-1, desmin, IL-6, CTGF, PDGF, and TIMP-1 were also significantly lower in the calcipotriol treatment group than those in the model group, while the expression of MMP-1 was significantly higher than that in the model group in isolated rat liver tissues (Figure 5(j)). Together, we firmly believe that the VDR-mediated calcipotriol treatment exerted an anti-liver fibrosis effect by regulating fibrosis-related factors *in vivo*.

## 4. Discussion

Liver fibrosis is a pathological process in which the dynamic balance of ECM is disturbed when the liver tissue is stimulated by various pathogenic factors. If the structure of liver lobules is destroyed on the basis of diffuse fibrosis, regenerating nodules appear and further form pseudo-lobules, resulting in cirrhosis. Liver fibrosis is an intermittent necessary stage of cirrhosis caused by different etiologies with complicated mechanisms, involving a variety of cellular inflammatory factors and complex cell biological behaviors.

In addition to the traditional regulation of calcium and phosphorus metabolism and bone metabolism, vitamin D and its receptor offer a wide range of physiological functions such as regulating cell proliferation, differentiation, and immune-inflammatory responses [7,8]. Vitamin D binds to the E region of the VDR structure and forms a heterodimer with the retinol X receptor (RXR_S_) [26], which then specifically recognizes the vitamin D response element (VDRE) on the promoter of the target gene in the nucleus to regulate the transcription process [27]. Numerous studies demonstrated that vitamin D/VDR was involved in the regulation of diabetes [28], chronic kidney disease [29], pulmonary fibrosis, chronic inflammation, and fibrosis of cardiovascular disease [30,31], along with the occurrence and development of many liver diseases [32]. Several researchers indicated that low levels of 25(OH)D3 and high levels of vitamin B12 were predictors of poor prognosis of liver disease [33]. Among them, several reports enunciated that low levels of 25(OH)D3 in serum could increase the risk of nonalcoholic fatty liver disease (NAFLD), and the decrease in serum 25(OH)D3 was associated with the degree of liver tissue steatosis, necrotizing inflammation, and fibrosis [34–36]. Similar to the above research results, we have observed that the widely distributed VDR in the cytoplasm and nucleus was expressed lesser in the severe liver fibrosis group compared to the mild liver fibrosis group. Similar studies have also shown that the expression of VDR in the subgroup of severe fibrosis was lower than that of the subgroup of mild fibrosis in genotype I HCV patients, and similar findings were found in patients with nonalcoholic fatty hepatitis (NASH) [37,38].

Notably, the damage of the liver often leads to the production of various cellular inflammatory factors, which in turn activates HSCs and transforms it into MFB, thereby starting to express a large number of musculo-skeletal proteins, such as α-SMA [39]. In this study, it was observed that the expression of α-SMA in the severe liver fibrosis group was significantly stronger than that in the mild liver fibrosis group. Moreover, the correlation analysis showed that the expression of VDR was negatively correlated with the degree of liver fibrosis and the expression of α-SMA and Col-1. It is speculated that active vitamin D/VDR may affect the synthesis of Col-1 through some mechanism. In this regard, several researchers demonstrated that VDR might be combined with RXR and VDR-interacting repressor (VDIR). In the instances of non-binding to the ligand 1,25(OH)2D3, it could upregulate the target genes and *vice versa* [40]. It could be inferred that, on the one hand, patients with chronic liver disease have vitamin D deficiency; on the other hand, the expression of VDR in the liver is down-regulated, resulting in insufficient VDR/RXR binding to the corresponding ligands. Thereby, binding the promoter of the type 1 collagen fiber gene through VDIR (1αnVDRE) leads to an increase in the expression of Col-1 protein and promotes the process of fibrosis. In this regard, several reports demonstrated that the isolated resting HSCs from the rats resulted in the highly expressed VDR, and the expression of VDR decreased after activation [24]. In addition, studies have found that calcipotriol could promote VDR gene transcription and protein expression in B16 cells [41]. Similarly, we applied calcipotriol to stimulate the LX-2, resulting in the increased expression of VDR significantly. Similarly, the above results were also verified in the CCl_4_-induced rat fibrosis model in this study.

After the establishment of LX-2 differentially expressing the VDR protein, it was observed that the cell proliferation ability of the calcipotriol group was increased after the VDR expression was increased, while the cell proliferation ability of the shVDR group was increased. Several investigations presented that, Kupffer cells in the liver could secrete TNF-α in large quantities in the instances of damaged liver cells and then initiate and maintain HSCs activation through the NF-κB pathway [4,5]. In some instances, TNF-α was applied to stimulate HSCs in rats and found that TNF-α promoted the proliferation of HSCs [42]. These observations were consistent with our results, implying that TNF-α stimulation and its mediated NF-κB pathway might have been involved in the inhibitory effect of VDR on HSCs activation. However, the mechanism is rarely reported in previous studies. In this study, we observed that after TNF-α stimulates LX-2, the expression of VDR was decreased, the cells continued to be activated, the expression of α-SMA was up-regulated, and Col-1 was produced in large quantities. Furthermore, in each group of LX-2 cells induced by TNF-α, the expression of nVDR was down-regulated, while the expression of NF-κB, p-IκBα, p-IKKβ, and p-p65 were up-regulated. Moreover, the expressions of NF-κB, p-IκBα, p-IKKβ, and p-p65 were up-regulated in the shVDR group and down-regulated in the calcipotriol group. These findings were consistent with the previous reports, and the reason might be that VDR and IKKβ could interact at the C-terminus, disrupting the formation of the IKK complex and blocking the p65/p50 nuclear translocation [10,43].

Our detection of the expression of fibrosis-related factors *in vitro* and *in vivo* models found that the expression trends of pro-fibrosis factors PDGF, TGF-β, desmin, IL-6, CTGF, and TIMP-1 are opposite to that of VDR, while the expression trends of anti-fibrosis factor MMP-1 consistent with VDR. The changes of these fibrosis-related factors reduced the proliferation and the ability of HSCs to secrete collagen fibers and enhanced the ability of cells to degrade collagen, and ultimately reduced the deposition of ECM [44]. Further detection of mitochondrial apoptosis pathway-related proteins resulted that the increase of LX-2 cell apoptosis was related to the enhancement of the mitochondrial apoptosis pathway. Apoptosis is an important metabolic pathway for the activation of HSCs, which is closely related to the occurrence and development of liver fibrosis [45]. Accordingly, an increase in the apoptosis rate of activated HSCs could be an important target for the prevention and treatment of liver fibrosis.

## 5. Conclusion

In conclusion, this study revealed the expression of VDR on HSCs and their ability to proliferate and apoptosis, secreting, and degradation of collagen fibers through the LX-2 cell model and the CCl_4_-induced fibrosis model of SD rats. Notably, this finding presents an important regulatory role and is an important entry point for anti-liver fibrosis. As a selective VDR agonist, calcipotriol also presents potential value in anti-liver fibrosis.

## Highlights

- VDR is differentially expressed in liver fibrosis patients.
- VDR regulates the function of HSCs with the participation of the NF-κB pathway.
- Calcipotriol could effectively reduce (CCl_4_)-induced liver fibrosis in the rat model.
